# Supplementary material for: From sequence to enzyme mechanism using multi-label machine learning
Source: BMC Bioinformatics. 2014 May 19;15:150. doi: 10.1186/1471-2105-15-150 (PMC4229970; doi:10.1186/1471-2105-15-150)
Supplement: Additional file 2 — Java code of ml2db. Additional file ml2db_code.tar.gz contains the Java source code to run the multi-label machine learning experiments and save the results to database. The code’s Javadoc is included. [file 1471-2105-15-150-S2.zip › additional file 2/ml2db/ml2db/doc/main/ml2db/al/utility/neighbours/NeighbourhoodNetwork.html]

NeighbourhoodNetwork


JavaScript is disabled on your browser.


- Overview
- Package
- Class
- Use
- Tree
- Deprecated
- Index
- Help

- Prev Class
- Next Class

- Frames
- No Frames

- All Classes

- Summary:
- Nested |
- Field |
- Constr |
- Method

- Detail:
- Field |
- Constr |
- Method


main.ml2db.al.utility.neighbours

## Class NeighbourhoodNetwork

- java.lang.Object
- - main.ml2db.al.utility.UtilityMetric
  - - main.ml2db.al.utility.neighbours.NeighbourhoodNetwork

- All Implemented Interfaces:
  :   java.io.Serializable

  ---

    

  ```
  public class NeighbourhoodNetwork
  extends UtilityMetric
  implements java.io.Serializable
  ```

  A collection of instances "needed" to label other instances.

  Version:
  :   21 Jul 2011

  Author:
  :   Luna De Ferrari ldf(at)st-andrews.ac.uk

  See Also:
  :   Serialized Form

- - ### Field Summary

    Fields

    | Modifier and Type | Field and Description |
    | --- | --- |
    | `static int` | `NUMBER_OF_NEIGHBOURS_1` |
  - ### Constructor Summary

    Constructors

    Constructor and Description

    `NeighbourhoodNetwork(IdMultiLabelInstances unlabelledInstances)`

    a list of needed instances
  - ### Method Summary

    Methods

    | Modifier and Type | Method and Description |
    | --- | --- |
    | `Neighbourhood` | `findBySparseAttribString(java.lang.String instanceString)` public static void main(String[] args) { int arffId = 13; String filePath = ProjectParameters.PREDICTIONS\_PATH + arffId + "\_plants" + TimeUtils.getTimeStampForFileName() + ".dot"; try { IdMultiLabelInstances plants = new IdMultiLabelInstances(arffId, ProjectParameters.getRemoteDbManagers()); NeighbourhoodNetwork network = new NeighbourhoodNetwork(plants); GraphVizPrinter .printNeighboursForGraphviz(filePath, network, false); } catch (InvalidDataFormatException e) { // TODO Auto-generated catch block e.printStackTrace(); } } |
    | `Neighbourhood` | `findByWekaHashCode(int hashCode)` |
    | `java.util.HashMap<java.lang.String,Neighbourhood>` | `getInstancesTree()` |
    | `java.lang.String` | `getMetricName()` |
    | `LinearNNSearchSkipInstanceItself` | `getNeighboursSearcher()` |
    | `IdMultiLabelInstances` | `getUnlabelledInstances()` |
    | `double` | `getUtility(mulan.classifier.MultiLabelLearner trainedLearner, IdInstance instance)` Only works with identified instances. |
    | `void` | `initialise()` |
    | `static void` | `main(java.lang.String[] args)` |
    | `int` | `numberOfUnlabelledNeedyInstances(java.lang.String instanceId)` |
    | `void` | `refresh(IdMultiLabelInstances labelled, IdMultiLabelInstances unlabelled, IdInstance instanceLabelled)` Refresh the set of labelled and unlabelled instances |
    | `void` | `setNeighboursAndNeedy()` |

    - ### Methods inherited from class main.ml2db.al.utility.UtilityMetric

      `getUnlabelled, setUnlabelled`
    - ### Methods inherited from class java.lang.Object

      `equals, getClass, hashCode, notify, notifyAll, toString, wait, wait, wait`

- - ### Field Detail


    - #### NUMBER\_OF\_NEIGHBOURS\_1

      ```
      public static final int NUMBER_OF_NEIGHBOURS_1
      ```

      See Also:
      :   Constant Field Values
  - ### Constructor Detail


    - #### NeighbourhoodNetwork

      ```
      public NeighbourhoodNetwork(IdMultiLabelInstances unlabelledInstances)
      ```

      a list of needed instances
  - ### Method Detail


    - #### findBySparseAttribString

      ```
      public Neighbourhood findBySparseAttribString(java.lang.String instanceString)
      ```

      public static void main(String[] args) { int arffId = 13; String filePath
      = ProjectParameters.PREDICTIONS\_PATH + arffId + "\_plants" +
      TimeUtils.getTimeStampForFileName() + ".dot";
      try { IdMultiLabelInstances plants = new IdMultiLabelInstances(arffId,
      ProjectParameters.getRemoteDbManagers()); NeighbourhoodNetwork network =
      new NeighbourhoodNetwork(plants); GraphVizPrinter
      .printNeighboursForGraphviz(filePath, network, false);
      } catch (InvalidDataFormatException e) { // TODO Auto-generated catch
      block e.printStackTrace(); }
      }


    - #### findByWekaHashCode

      ```
      public Neighbourhood findByWekaHashCode(int hashCode)
      ```


    - #### getInstancesTree

      ```
      public java.util.HashMap<java.lang.String,Neighbourhood> getInstancesTree()
      ```


    - #### getMetricName

      ```
      public java.lang.String getMetricName()
      ```

      **Specified by:**
      :   `getMetricName` in class `UtilityMetric`


    - #### getNeighboursSearcher

      ```
      public LinearNNSearchSkipInstanceItself getNeighboursSearcher()
      ```


    - #### getUnlabelledInstances

      ```
      public IdMultiLabelInstances getUnlabelledInstances()
      ```


    - #### getUtility

      ```
      public double getUtility(mulan.classifier.MultiLabelLearner trainedLearner,
                      IdInstance instance)
      ```

      Only works with identified instances.

      **Specified by:**
      :   `getUtility` in class `UtilityMetric`


    - #### initialise

      ```
      public void initialise()
      ```

      **Specified by:**
      :   `initialise` in class `UtilityMetric`


    - #### numberOfUnlabelledNeedyInstances

      ```
      public int numberOfUnlabelledNeedyInstances(java.lang.String instanceId)
      ```


    - #### refresh

      ```
      public void refresh(IdMultiLabelInstances labelled,
                 IdMultiLabelInstances unlabelled,
                 IdInstance instanceLabelled)
      ```

      **Description copied from class: `UtilityMetric`**

      Refresh the set of labelled and unlabelled instances

      **Specified by:**
      :   `refresh` in class `UtilityMetric`


    - #### setNeighboursAndNeedy

      ```
      public void setNeighboursAndNeedy()
      ```


    - #### main

      ```
      public static void main(java.lang.String[] args)
      ```


- Overview
- Package
- Class
- Use
- Tree
- Deprecated
- Index
- Help

- Prev Class
- Next Class

- Frames
- No Frames

- All Classes

- Summary:
- Nested |
- Field |
- Constr |
- Method

- Detail:
- Field |
- Constr |
- Method
